# Approach to sexuality among older adults for Primary Health Care professionals

**DOI:** 10.1590/0034-7167-2024-0199

**Published:** 2025-06-20

**Authors:** Fernanda Alencar de Almeida Pereira Fabrício, Gilka Paiva Oliveira Costa, Ana Mabel Sulpino Felisberto, Maria Adelaide Silva Paredes Moreira, Antônia Lêda Oliveira Silva, Josiane Maria Oliveira de Souza

**Affiliations:** I Universidade Federal da Paraíba. João Pessoa, Paraíba, Brazil; II Universidade de Brasília. Brasília, Distrito Federal, Brazil

**Keywords:** Aged, Sexuality, Health Personnel, Care, Primary Health Care., Anciano, Sexualidad, Personal de Salud, Atención, Atención Primaria de Salud.

## Abstract

**Objectives:**

to reveal Primary Health Care professionals’ experience regarding the approach to sexuality among older adults.

**Methods:**

this is exploratory research with a qualitative approach, carried out with 40 healthcare professionals, including physicians and nurses, who participated in a semi-structured interview. The interviews were recorded and analyzed using the thematic content analysis technique.

**Results:**

the data were analyzed and grouped into five empirical categories: Descriptions of sexuality; Difficulties in addressing sexuality; Importance of addressing sexuality among older adults; Professional indicated to address sexuality; and Older adults’ perceptions about sexuality according to healthcare professionals.

**Final Considerations:**

the study demonstrated that professionals face difficulties in addressing sexuality among older adults, which reinforces the stereotype of asexuality. Therefore, it is necessary to seek to address this issue in care planning actions and seek to overcome related cultural and social barriers.

## RESUMEN

**Objetivos:**

revelar la experiencia de profesionales de la Atención Primaria de Salud en relación al abordaje de la sexualidad entre personas mayores.

**Métodos:**

se trata de una investigación exploratoria con enfoque cualitativo, realizada con 40 profesionales de la salud, entre médicos y enfermeras, que participaron en una entrevista semiestructurada. Las entrevistas fueron grabadas y analizadas mediante la técnica de análisis de contenido temático.

**Resultados:**

los datos fueron analizados y agrupados en cinco categorías empíricas: Descripciones de sexualidad; Dificultades para abordar la sexualidad; Importancia de abordar la sexualidad entre las personas mayores; Profesional indicado para abordar la sexualidad; y Percepciones de las personas mayores sobre la sexualidad según los profesionales de la salud.

**Consideraciones Finales:**

el estudio demostró que los profesionales enfrentan dificultades para abordar la sexualidad entre las personas mayores, lo que refuerza el estereotipo asexual. Por lo tanto, es necesario buscar abordar este tema en las acciones de planificación del cuidado y buscar superar las barreras culturales y sociales relacionadas.

## INTRODUCTION

The World Health Organization (WHO) defines active aging as a health policy aimed at improving older adults’ health, participation and safety. The WHO advocates that governments, international organizations and civil society implement programs and policies to promote active and healthy aging. In this regard, it is up to healthcare professionals to lead the challenges of healthy aging from a broader health perspective, involving intersectoral and transdisciplinary actions, which seek, among other things, to eliminate age discrimination and promote recognition of the diversity of older populations^(1)^.

With the phenomenon of population aging on the rise, the 21^st^ century is notable for the significant growth of the elderly population worldwide, which is increasing by around 3% per year^(2)^. According to the WHO, in 2022, the world population over 60 years old increased from 841 million and is expected to reach 2 billion by 2050. In this context, Brazil is facing a rapid aging process, with an estimate that the elderly population will account for 30% of the population by 2050^(3)^.

In the most recent Continuous Brazilian National Household Sample Survey, between 2012 and 2021, a sharp decrease in the fertility rate and a significant increase in the aging of the Brazilian population were observed. The number of people under 30 years of age decreased by 5.4%, while the share of people aged 60 or over went from 11.3% to 14.7% of the population, growing from 22.3 million to 31.2 million people, which accounts for an increase of 39.8%^(4)^.

Sexuality is a fundamental component of the human life cycle, a dimension related to health, being an important component of life and recognized as a basic need, fundamental for maintaining relationships between individuals. Therefore, sexual activity in old age is seen as necessary and even beneficial for older adults’ health, being an important indicator for the quality of life among this population^(5-8)^.

Thus, sexuality must be understood as an essential element at any time in life, and must be interpreted as a multifactorial process that encompasses a network of meanings, being the result of personal, intimate and singular experience^(9)^. The construction of sexuality occurs throughout life, including, in addition to physical aspects, psychological, cultural, interactional, self-image aspects, among others^(10-13)^. Studies suggest that a considerable number of older adults continue to express sexual interest, even as they age, and their expression can be empowering, affirmative, joyful and positive. Therefore, discussing the topic of sexuality with this population, in healthcare services, is extremely important to demystify the idea that as people grow older they become asexual^(11,14)^.

However, the topic of sexuality in aging is still surrounded by prejudice, both on by younger people and older adults themselves, as well as many healthcare professionals. This is a topic in gerontology that needs to be addressed more. There are several cultural myths and common beliefs in society that discriminate against older adults who express sexual desires and seek sexual fulfillment^(12,15)^. Such prejudice influences aging policies and scientific research on this topic, making it important to understand that sexuality goes beyond sexual desires, and is also a matter of health and strengthening personal bonds^(5,16)^.

Scientific evidence suggests that little is known about how healthcare professionals deal with this topic and/or perceive and manage older adults’ sexual concerns. Despite the importance of sexuality for quality of life, many professionals neglect the topic in health contexts, often underestimating it^(17)^. Sexuality in older adults’ lives should be seen as natural, pleasurable and healthy, promoting the well-being of those who practice it. Understanding what happens is a constructive strategy in combating socially ingrained myths and stereotypes^(18)^.

In this regard, despite the strong association between sexuality and quality of life, and the undeniable health benefits, caring for sexual health during aging is still a topic little explored among healthcare professionals. Given this, the question is: what do healthcare professionals think about the need to address sexuality with older adults during care?

## OBJECTIVES

To reveal Primary Health Care (PHC) professionals’ experience regarding the approach to sexuality among older adults.

## METHODS

### Ethical aspects

In this study, the ethical aspects relevant to research involving human beings were considered, in accordance with Resolution 466/12^(19)^, and were approved by the *Universidade Federal da Paraíba* Research Ethics Committee. The Informed Consent Form was obtained from all individuals involved in the study through a written document. To maintain anonymity, participants’ statements were identified throughout the text with the letter “P”, representing healthcare professionals, followed by a number corresponding to the order of the statements (P01... P40).

### Study design

This is exploratory research with a qualitative approach, which focuses on understanding the human experience as lived, through the careful collection and analysis of narrative and subjective materials^(20)^. The study was also based on the COnsolidated criteria for REporting Qualitative research (COREQ), recommended for research reports conducted through interviews or focus groups, with well-defined and structured criteria in three domains: research team characterization and qualification; study design; and analysis of results^(21)^.

### Study setting

This research was carried out in the Basic Health Units (BHUs) of the municipality of João Pessoa, Paraíba, between October 2018 and April 2019. The municipality has 93 BHUs and 203 Family Health teams (FHt) in its territory^(22)^.

### Data source

Twenty physicians and 20 nurses were interviewed based on the following inclusion criteria: providing direct assistance to older adults in PHC at the time of data collection and agreeing to participate in the research. The sample was therefore a convenience sample.

### Data collection and organization

Data collection was carried out through recorded semi-structured interviews, previously scheduled with healthcare professionals. The interviews were conducted in a private room at the BHU itself, outside participants’ work shift, to ensure confidentiality and minimize interference. Each interview lasted an average of 30 minutes. The collection instrument consisted of two parts: the first explored participants’ sociodemographic data, such as age, sex, profession and time of experience; the second part addressed issues related to the approach to sexuality for older adults in the FHt daily care.

The research was completed considering the time interval proposed for data collection, the numerical parity between the two professional classes, material quality, the absence of new information and, consequently, data saturation.

### Data analysis

The data collected in the interviews were analyzed using the content analysis technique proposed by Bardin^(23)^, following the following stages: constitution of the *corpus* formed by the 40 interviews, using the exhaustiveness criterion; material skimming and preparation (transcription and full reading of recorded interviews); coding (excerpts were made by semantic level, using thematic unit as recording unit, with the identification of interviewees by the number P01-40); categorization, which followed the semantic logic and in accordance with the objective of the study.

After applying the technique, the results revealed five thematic categories, namely: Descriptions of sexuality; Difficulties in addressing sexuality; Importance of addressing sexuality among older adults; Professional indicated to address sexuality; and Older adults’ perceptions about sexuality according to healthcare professionals.

Grouping and categorization were validated by three different researchers with expertise in the content analysis technique and interpretation of results.

## RESULTS

Forty healthcare professionals participated in the research, 20 nurses and 20 physicians, with an average age of 47 years (minimum 25 and maximum 70 years) and time in the profession of 20 years (minimum 1 and maximum 46 years).

The results obtained from content analysis of interviews with physicians and nurses working in PHC indicated five categories on how they approach sexuality with older adults.

### Category I - Descriptions of sexuality

This category was formed by the analysis units in which healthcare professionals (physicians and nurses) talk about sexuality when providing care to older adults. It can be seen that the approach can be initiated by both healthcare professionals and older adults.

In this study, it was observed that the approach to sexuality does not occur systematically, being mentioned especially during the cytopathological exam collection, carried out by nursing professionals. Physicians, on the other hand, did not mention this practice in their statements.

Another time when the topic of sexuality can be addressed is during group meetings, as mentioned by some nurses and physicians. Global awareness campaigns, such as “Pink October” and “Blue November”, which focus on preventing breast and prostate cancer, were highlighted by these professionals as opportunities to discuss issues related to sexuality among older adults.

The approach (...) *neither frequent nor occasional* (...) was cited by few physicians and nurses, such as:

[...] *patients come with these complaints. We should ask them, but in practice, they bring up the subject themselves, so we should ask, but that doesn’t happen* [...]. (P1)

[...] *in the service, the approach is made during the cytology. Older adults try to talk about the difficulty in the sexual act* [...]. (P4)

[...] *the demand almost always comes from him* [...]. (P22)

[...] *we don’t talk about this topic, it’s a lot of work and difficult, there’s a lot of prejudice, especially older adults think they shouldn’t have it* [...]. (P24)

[...] *I don’t approach them much. They complain more than we ask* [...]. (P25)

However, in individual consultations, most physicians and nurses reported that sexuality is only discussed at the request of the older adults themselves. It is noteworthy that some professionals, both in nursing and medicine, stated that they do not discuss sexuality at any time. From older adults’ perspective, it is common for them to talk about sexuality among themselves; however, this conversation occurs occasionally, according to few nurses and physicians. Others mentioned that this approach usually occurs during the cytopathological exam collection.

### Category II - Difficulties in approaching sexuality

This category consists of units of analysis in which healthcare professionals describe challenges faced when talking about sexuality with older adults. Thus, they mention several obstacles, such as lack of training and adequate qualifications, in addition to the absence of appropriate materials to help address the topic.

Feelings of discomfort, prejudice and embarrassment can be observed in statements, both by older adults and healthcare professionals, in dealing with the topic:

[...] *I have difficulty with this subject. Training would be good to guide us, give us direction, whether we are right, wrong, how to approach it in the best way* [...]. (P7)

[...] *it would be important to have material, training. Few people report it, most don’t even talk about it, men don’t look for it very often* [...]. (P13)

[...] *there is a taboo on the subject, but I try to ask naturally, I think there is a lack of training to be more natural, a more natural approach* [...]. (P27)

[...] *there was no training in the undergraduate course to talk about this subject* [...]. (P40)

It is observed that, when talking about social prejudice, cultural and generational differences, professionals add the perception that the topic is not relevant in routine consultations, especially when compared to health issues considered more urgent. Moreover, there is the presence of personal beliefs of some professionals that influence the approach to the topic, making it difficult to discuss sexuality in care.

According to professionals, older adults feel “discomfort, prejudice, taboo and embarrassment” when addressing the subject, according to reports from both physicians and nurses. This proves to be one of the main obstacles to addressing sexuality during care.

Professionals reported that many older adults avoid talking about the topic because they feel embarrassed or because they believe that sexuality should not be discussed in their age group. This discomfort, according to professionals, is aggravated by cultural prejudices that associate sexuality with youth, making older adults feel inappropriate or judged when talking about the subject:

[...] *I think there are difficulties, especially for older adults, in opening up and talking* [...]. (P4)

[...] *there are still barriers, they don’t speak so naturally* [...]. (P18)

[...] *there is a lot of prejudice, especially from older adults. It ends up being difficult to approach* [...]. (P19)

[...] *since they have a taboo, it is still a closed subject for them. It turns out that they do not protect themselves* [...]. (P21)

[...] *I think there is still a taboo, especially in relation to older adults for professionals on the subject* [...]. (P25)

[...] *I perceive prejudice on their part. I don’t see any difficulties on my part* [...]. (P39)

It was noted that professionals emphasized that “embarrassment” can create barriers in communication, making it difficult to create a safe and welcoming environment for older adults to express their sexual concerns or needs. Some professionals reported that, even when they tried to introduce the topic, they often encountered resistance or refusal to talk as a way to avoid causing further discomfort to older adults.

In interviewees’ statements, units of analysis are identified in which they highlight the “prejudice” by society in relation to the topic of sexuality in old age, considered difficult to approach professionally and a social stigma. This aspect is configured as one of the main barriers to this approach, even in a natural and open way, during care for older adults.

It is known that society still imposes cultural restrictions by associating sexual practice with the younger stages of life, reinforcing the idea that older adults should not or cannot have an active sex life:

[...] *there is a lot of prejudice, we also don’t know how to deal with it and approach it properly without hurting the principles of some* [...]. (P4)

[...] *we live in a country of taboos, the approach is difficult, there is a taboo on the subject* [...]. (P22)

[...] *I think it needs to be more natural* [...]. (P27)

Professionals feel more uncomfortable talking about sexuality, both for themselves and for older adults, especially when interaction occurs between professionals and older adults of different sexes. This aspect can inhibit conversation, especially when the older adult is a woman and the healthcare professional is a man, or vice versa. Some interviewees reported that older adults prefer to discuss sexuality issues with female nursing professionals, as they feel more comfortable. On the other hand, men may have difficulty discussing sexuality with professionals of the opposite sex, especially when dealing with issues related to sexual impotence:

[...] *I don’t feel comfortable talking about the subject with older adult men* [...]. (P6)

[...] *women find it more difficult to talk to male physicians* [...]. (P31)

[...] *they are more embarrassed, because I think that, as I am a woman, they are embarrassed to talk about the subject* [...]. (P38)

[...] *I think that, because I’m younger, I had difficulty both being natural about the subject and I noticed difficulties on their part. It turns out that they don’t complain to me* [...]. (P15)

[...] *the subject is uncomfortable. I’m from a generation that is very difficult to talk about, not only for older adults* [...]. (P29)

[...] *there is no difficulty, none, neither on the part of the professionals nor the older adults. The older adults participate, in a good way, in the dynamics, total acceptance* [...]. (P5)

[...] *I have no difficulty talking about this approach because of the way I am, I am very playful, we open the subject and they say they no longer feel like it or that they cannot do it* [...]. (P37)

Thus, some nurses reported that there was no difficulty in mentioning whether an update was necessary, in agreement with some physicians. Nurses highlighted a concern with the intergenerational difference, an aspect that was not identified in physicians’ statements.

### Category III - Importance of addressing sexuality among older adults

Professionals highlighted the relevance of the topic and the need to address sexuality among older adults more frequently. Hence, all nurses emphasized the importance of addressing sexuality for older adults, which should be included in the routine. Other professionals expressed that, although they found the topic important, the approach should occur in groups, lectures, campaigns or when requested by older adults. Physicians considered it relevant to address the topic, however, like the nurses, most mentioned that the approach should be routine, while some understood that it would not be important. It was observed that there was no consensus among professionals:

[...]*yes, because they are even more sad without a sex life. It’s a sum. For those of us who understand, it affects us, imagine for those who don’t* [...]. (P11)

[...] *I think it’s very important. Today, the rate of STIs has increased a lot. They are embarrassed to use condoms. They take a long time to come to us when they have symptoms, and we have older adults with sexually transmitted diseases, and they should be routinely assessed* [...]. (P12)

[...]. *I think it’s interesting to open up more about this subject, as there may be several problems that are being hidden* [...]. (P13)

[...] *yes, too important, it should be routine. We need to ask this question* [...]. (P26)

[...] *very important, it is part of life, erection problems, anxiety, it ends up becoming a snowball. When addressed, it improves mental health* [...]. (P33)

The difference between sexes was also mentioned by interviewees as a barrier in addressing sexuality among older adults. Physicians and nurses reported that men and women often have different levels of openness and comfort when talking about their sexuality, which can make it difficult to approach the topic. According to professionals, men tend to address issues related to sexual performance more directly, while women, when mentioning the topic, tend to focus on emotional aspects or lack of desire.

### Category IV - Professionals indicated to address sexuality

In this category, interviewees indicated professionals best suited to talk about sexuality with older adults during healthcare. The majority of nurses emphasized that all healthcare professionals should approach the subject. To this end, they suggested the need to train professionals to provide care. Only two reported that this responsibility falls to physicians, nurses and psychologists. As for physicians, it was observed that 11 stated that all healthcare professionals should talk about sexuality; eight indicated physicians, nurses and psychologists; and only one interviewee mentioned that only physicians should talk about sexuality with older adults:

[...] *the entire team of professionals, if they have knowledge and do not make the subject vulgar* [...]. (P3)

[...] *the entire team should be trained, including health agents, as there are many older adults who have a connection with the agents, and they bring complaints and problems* [...]. (P5)

[...] *my opinion is that everyone should be trained to do so, so as not to undo what is being worked on: physicians, nurses, dentists, nursing technicians, health agents* [...]. (P23)

[...] *every team must address* [...]. (P25)

It was found that professionals pointed out the lack of time in consultations as a relevant obstacle, since many professionals consider that there is little space for discussions about sexuality in a service focused mainly on physical and chronic issues. Most professionals reported facing difficulties in dealing with the topic, justifying the lack of training, qualification and material resources as a significant barrier to addressing sexuality with older adults.

### Category V - Older adults’ perceptions about sexuality according to healthcare professionals

In this category, we can observe units of analysis of professionals addressing the importance of talking about sexuality with older adults during healthcare. Professionals report that older adults show concern about “reduced libido”, as expressed in “lack of desire” during consultations:

[...] *older adults say that their husbands want it and they don’t want it anymore* [...] *they don’t feel like it, or the opposite, they want it and they don’t want it.* (P2)

[...] *they think people find it ridiculous for them to talk about sex.* (P12)

[...] *think they shouldn’t talk openly about the subject* [...]. (P15)

[...] *they complain a lot about the aging process, the difficulties, the dryness* [...]. (P18)

[...] *they apologize, they are very embarrassed, they make a detour to talk. They think that the desire for sex is wrong, that she is naughty, they already say that they are embarrassed* [...]. (P21)

*They think they are too old* [...]. (P29)

*Many older adults are already talking about dyspareunia, the difficulties of menopause that end up getting in the way of their sex life* [...]. (P33)

There is a concern among those interviewed about the “prejudice” and “difficulties” associated with the aging process with regard to older adults’ sexuality.

## DISCUSSION

The results revealed the prominent difficulty in addressing the issue of sexuality among older adults in the care provided by healthcare professionals working in PHC, with emphasis on difficulties related to professional training, personal values, intergenerational conflicts and the social construction of older adults as asexual.

Such difficulties were reported in another study involving PHC professionals who presented conservative positions in relation to sexuality among older adults, even demonstrating knowledge on the subject^(24)^. It is worth noting that the difficulties encountered in this study, such as lack of knowledge and training on the subject, lack of time for consultations and discomfort generated by the subject, were also identified in other related studies^(8,11,18,25)^.

The healthcare professionals interviewed revealed that addressing the topic of sexuality among older adults is difficult for most of them: 80% of physicians reported these difficulties, as did 70% of nurses. When analyzing the main difficulties, it is worth noting that 57.14% of nurses and 50% of physicians mentioned discomfort, prejudice and embarrassment by older adults as difficulties. This finding was corroborated by a study carried out in Belém-PA, in 2017, on healthcare professionals’ perception regarding sexuality in old age, which indicated that the greatest difficulties encountered when addressing the topic was “resistance from older adults”, as reported by 50% of those interviewed^(26)^.

Regarding the difficulties related to the lack of training, preparation and qualification in the subject, these were mentioned by 57.14% of nurses and 37.5% of physicians in this survey. These obstacles were also reported in a study with resident physicians in sexual and reproductive health in the community, as well as resident physicians in obstetrics and gynecology in England, who indicated that the main barriers to addressing sexual health in (peri)menopause by physicians were the discomfort perceived by patients and the lack of training in the curriculum. This study also mentioned the lack of time as a main barrier, which was cited by only one nurse interviewed in this survey^(14)^.

Lack of training was also mentioned in research conducted in São Paulo, which indicated that most physicians are unable to address the issue of sexuality in their older adult patients with chronic pain due to feelings of technical ineptitude as well as lack of time and fear of embarrassing the patient^(8)^. A study conducted with nursing students showed that older adults’ sexuality is seen as something negative and labeled as stereotypes. The statements in the study indicate a greater difficulty in approaching the subject with older adults, as a result of a culture that labels them as asexual and the deficient training of healthcare professionals^(26)^.

Another difficulty mentioned in this study was the difference between sexes, reported by 16.6% of physicians and 7.14% of nurses interviewed. This perception is corroborated by another survey conducted with healthcare professionals, in which interviewees identified sex as a factor that interferes with the discussion of sexuality, with many men feeling uncomfortable talking about sex with a female healthcare professional^(24)^. Furthermore, in another study, female older adults reported feeling discomfort when undergoing gynecological examinations with male professionals, which generates great insecurity and discouragement in seeking out such professionals^(27)^.

As for the timing of addressing the issue of sexuality, physicians and nurses emphasize that they do so during individual care. However, most nurses (65%) reported that they address the issue during the cervical cytology exam collection. However, a recent Brazilian study highlighted that nursing professionals talk little about the issue and need to address sexuality without judgment, according to older adults, since there are different ways of approaching sexuality during the aging process^(27)^.

In this sense, it is necessary to emphasize the importance of professionals being more attentive and prepared to offer support to older adults on the topic of sexuality, as demonstrated in other studies^(24,27,28)^. They must free themselves from taboos, prejudices and criticism, adopting more appropriate positions on this topic, given that there is still a conservative stance on sexuality and a lack of preparation among healthcare professionals^(17,27,28)^.

The interview with physicians showed that sexuality is discussed occasionally during consultations, with priority given to older adults. This approach, although insufficient, is also observed in other studies, such as the one conducted by Oxford^(12)^, which found that 42.5% of geriatricians never asked about their patients’ sexual history and 57.5% did so only occasionally. In a recent study conducted with 596 older adults in the five Brazilian regions, it was found that 77.0% of participants never received guidance on sexuality from healthcare professionals^(25)^. Another Brazilian study supports this reality, since only 17% to 32% of Brazilian men and 19% to 29% of Brazilian women received any guidance on sexuality from medical professionals^(29)^.

Therefore, by waiting for older adults to show interest in addressing their sexuality, many opportunities are lost. The literature indicates that, even in the presence of dysfunctions that seriously affect quality of life, most feel uncomfortable or reluctant to discuss sexuality with healthcare professionals, especially if they have to initiate the issues on their own^(18,24)^. Furthermore, a study reveals that older adults perceive that healthcare professionals are not interested in or do not understand the sexuality of this age group and, therefore, avoid initiating discussions on the topic^(24)^. In contrast, another study shows that older adults who received some guidance on sexuality from healthcare professionals experience life and sexual intercourse better^(30)^.

Although most geriatricians approach the topic occasionally, all nurses and 95% of physicians reported that it is important to address the issue. This similarity was also observed in a 2011 study in England, which showed that 96.7% of geriatricians consider it important to manage sexual problems^(12)^.

Nursing and medical teams, in the professional category indicated to address sexuality, attributed to all healthcare professionals the need to proceed with the approach and to be trained on the subject. A study supported this idea by demonstrating that all healthcare providers, including psychologists, physicians (such as those in primary care, gynecology, urology, primary care and oncology), social workers, nurses, pharmacists, occupational therapists, health educators, directors of homes for older adults and nutritionists, can potentially participate in modulating positions of older adults regarding their own sexual lives, both directly and indirectly^(17)^.

Lack of communication with healthcare professionals not only results in poor treatment management, but also increases the risk of sexually transmitted infections (STIs). This was evidenced in a cross-sectional study (n=101) with older adults, in which 90% of respondents reported never having received information about the human immunodeficiency virus (HIV) and 80% had not received education about STIs. As a result, older adults may believe that they are not at risk of contracting a sexually transmitted disease. Therefore, there is a clear need to improve communication between professionals and older adults, as it was observed that professionals still maintain conservative positions regarding the topic of sexuality, in addition to demonstrating negative positions^(24)^.

This study demonstrates that an important area of healthcare remains neglected, with literature indicating that sexuality is fundamental to the well-being of everyone, including older adults^(25)^. People need to be educated about the changes that occur in sexual functioning as they age as well as the options available to help them. Furthermore, healthcare professionals need to be trained to raise awareness about sexuality in aging and improve their communication skills^(18)^. The challenge is to develop strategies that allow the approach to sexuality to occur naturally as part of older adults’ healthcare.

### Study limitations

The limitations of this research are that the results are based on the knowledge of only 40 healthcare professionals working in a single municipality in the Brazilian Northeast. The sample is relatively small and restricted to a single location, which limits data representativeness. Therefore, it is important to conduct studies with a larger number of professionals from different health units to enable more robust and accurate generalizations.

### Contributions to nursing and health

Exploring sexuality among older adults in the current scenario, characterized by the increasing life expectancy of the Brazilian and global population, makes a significant contribution to demystifying and redefining concepts that are deeply rooted in society, such as the idea that older adults are asexual. These concepts directly impact the quality of life of these people, influencing their mental and physical health.

The study can alert managers and healthcare professionals, especially those in PHC - the gateway to the Brazilian health system - about the need to include this topic in their training and care, both in groups and individually.

Healthcare professionals, including nurses, must prepare for the reality of population aging and address sexuality when caring for older adults. This approach can help break down cultural and intergenerational barriers, in addition to promoting more qualified, active, comprehensive, and welcoming care. This study seeks to bring these reflections to PHC professionals’ practice and other areas of the healthcare network, aiming to encourage greater reflection and dissemination on this important topic.

## FINAL CONSIDERATIONS

This study sought to reveal PHC professionals’ experiences in addressing sexuality with older adults, demonstrating that the approach is carried out occasionally and without proactivity. Healthcare professionals revealed that, when the approach takes place, discomfort linked to prejudice and lack of information involving the topic of sexuality among older adults is perceived. In addition, professionals highlight their lack of training to deal with issues related to sexuality.

The lack of communication between healthcare professionals and older adults neglects their needs and favors the acceptance of asexual stereotypes and the naturalization of sexuality as an undesirable subject, strengthening the barriers to assistance on this topic in the aging process.

In this sense, the approach to sexuality in PHC for older adults must be more inclusive and informed, since healthcare professionals play a vital role in demystifying stereotypes associated with older adults’ sexuality and promoting a healthy and satisfying sexual life. This paradigm shift not only benefits older adults, but also enriches care and strengthens the relationship between professionals and patients.

Recommendations to minimize such conceptions include the need for more dialogue about sexuality among healthcare professionals, aiming at a better understanding of older adults’ needs, in addition to avoiding neglect in relation to sexual and emotional health, as well as the lack of support in the experience of sexuality. Therefore, it is essential to observe strategies that minimize stereotypes by naturalizing the topic among older adults, seeking to break down significant barriers in healthcare. Overcoming these challenges requires the implementation of actions aimed at training healthcare professionals to approach sexuality in a more open and respectful way, recognizing the importance of this aspect in older adults’ lives.
