# Supplementary material for: Approach to sexuality among older adults for Primary Health Care professionals
Source: Rev Bras Enferm. 2025 Jun 20;78(2):e20240199. doi: 10.1590/0034-7167-2024-0199 (PMC12185140; doi:10.1590/0034-7167-2024-0199)
Supplement: Translation

# Translation: Abordagem da sexualidade entre pessoas idosas para os profissionais da Atenção Primária à Saúde

**DOI:** 10.1590/0034-7167-2024-0199pt

**Authors:** Fernanda Alencar de Almeida Pereira Fabrício, Gilka Paiva Oliveira Costa, Ana Mabel Sulpino Felisberto, Maria Adelaide Silva Paredes Moreira, Antônia Lêda Oliveira Silva, Josiane Maria Oliveira de Souza

**Affiliations:** I Universidade Federal da Paraíba. João Pessoa, Paraíba, Brasil; II Universidade de Brasília. Brasília, Distrito Federal, Brasil

**Keywords:** Idoso, Sexualidade, Profissional de Saúde, Atendimento, Atenção Primária à, Saúde.

## Abstract

**Objetivos:**

desvelar a vivência dos profissionais da Atenção Primária à Saúde sobre abordagem da sexualidade entre as pessoas idosas.

**Métodos:**

trata-se de pesquisa exploratória de abordagem qualitativa, realizada com 40 profissionais de saúde, entre médicos e enfermeiros, que participaram de uma entrevista semiestruturada. As entrevistas foram gravadas e analisadas por meio da técnica de análise de conteúdo temática.

**Resultados:**

os dados foram analisados e agrupados em cinco categorias empíricas: Descrições sobre sexualidade; Dificuldades para abordar a sexualidade; Importância de abordar a sexualidade entre os idosos; Profissional indicado para abordar a sexualidade; e Percepções dos idosos sobre a sexualidade segundo os profissionais de saúde.

**Considerações Finais:**

o estudo demonstrou que os profissionais enfrentam dificuldades para abordar a sexualidade entre as pessoas idosas, o que reforça o estereótipo de assexuado. Portanto, faz-se necessário buscar pautar essa temática nas ações de planejamento da assistência e buscar superar as barreiras culturais e sociais relacionadas.

## INTRODUÇÃO

A Organização Mundial da Saúde (OMS) define o envelhecimento ativo como uma política de saúde voltada para melhoria da saúde, participação e segurança das pessoas idosas. A OMS defende que os governos, as organizações internacionais e a sociedade civil implementem programas e políticas para promover o envelhecimento ativo e saudável. Nesse sentido, cabe aos profissionais de saúde liderar os desafios do envelhecimento saudável em uma perspectiva de saúde mais ampla, envolvendo ações intersetoriais e transdisciplinares, que busque, entre outras coisas, eliminar a discriminação de idade e promova o reconhecimento da diversidade das populações mais velhas^(1)^.

Com o fenômeno do envelhecimento populacional em plena ascensão, o século XXI se destaca pelo crescimento expressivo da população idosa em todo o mundo, que aumenta cerda de 3% ao ano^(2)^. Segundo a OMS, em 2022, a população mundial com mais de 60 anos passou de 841 milhões e deverá alcançar 2 bilhões até 2050. Neste contexto, o Brasil enfrenta um rápido processo de envelhecimento, com estimativa de que a população idosa representará 30% da população até 2050^(3)^.

Na mais recente Pesquisa Nacional por Amostra de Domicílios Contínua, entre 2012 e 2021, observou-se uma acentuada diminuição da taxa de fecundidade e um aumento significativo no envelhecimento da população brasileira. O número de pessoas com menos de 30 anos de idade diminuiu 5,4%, enquanto a parcela de pessoas com 60 anos ou mais passou de 11,3% para 14,7% da população, crescendo de 22,3 milhões para 31,2 milhões de pessoas, o que representa aumento de 39,8%^(4)^.

A sexualidade é um constituinte fundamental no ciclo da vida humano, uma dimensão relacionada à saúde, sendo um componente importante da vida e reconhecida como uma necessidade básica, fundamental para manutenção das relações entre os indivíduos. Sendo assim, a atividade sexual na terceira idade é vista como necessária e até mesmo benéfica para a saúde das pessoas idosas, sendo um indicador importante para a qualidade de vida entre esta população^(5-8)^.

Neste sentido, a sexualidade deve ser entendida como um elemento essencial em qualquer época da vida, e deve ser interpretada como um processo multifatorial que engloba uma rede de significados, sendo fruto da experiência pessoal, intimista e singular^(9)^. A construção da sexualidade ocorre ao longo da vida, compreendendo, além dos aspectos físicos, os aspectos psicológicos, culturais, interacionais, de autoimagem, entre outros^(10-13)^. Estudos sugerem que parte considerável das pessoas idosas continua a manifestar interesse sexual, mesmo com o avançar da idade, e sua expressão pode ser fortalecedora, afirmativa, alegre e positiva. Portanto, falar sobre a temática da sexualidade com esta população, no atendimento dos serviços de saúde, é de extrema importância para desmistificação da ideia de que, ao envelhecer, a pessoa se torna assexuada^(11,14)^.

Entretanto, o tema da sexualidade no envelhecimento ainda é cercado de preconceito, tanto por parte dos mais jovens quanto por parte das próprias pessoas idosas e de muitos profissionais de saúde, sendo um tema na gerontologia que necessita ser mais abordado. Existem diversos mitos culturais e crenças comuns na sociedade que discriminam o idoso que manifesta desejos sexuais e busca realização sexual^(12,15)^. Tal preconceito influencia as políticas de envelhecimento e as investigações científicas sobre essa temática, tornando-se importante compreender que a sexualidade vai além dos desejos sexuais, sendo também uma questão de saúde e fortalecimento dos vínculos pessoais^(5,16)^.

As evidências científicas sugerem que pouco se sabe sobre como os profissionais de saúde lidam com esse tema e/ou percebem e gerenciam as preocupações sexuais das pessoas idosas. Apesar da importância da sexualidade para a qualidade de vida, muitos profissionais negligenciam o tema nos contextos de saúde, frequentemente subestimando-os^(17)^. A sexualidade na vida da pessoa idosa deve ser vista de forma natural, prazerosa e saudável, promovendo o bem-estar daqueles que a praticam. Compreender o que acontece é uma estratégia construtiva no combate aos mitos e estereótipos socialmente arraigados^(18)^.

Neste sentido, apesar da forte associação entre sexualidade e qualidade de vida, e dos indiscutíveis benefícios para a saúde, o cuidado com a saúde sexual durante o envelhecimento ainda é um tema pouco explorado entre os profissionais de saúde. Diante disso, questiona-se: o que pensam os profissionais de saúde sobre a necessidade de abordar a sexualidade com pessoas idosas durante o atendimento?

## OBJETIVOS

Desvelar a vivência dos profissionais da Atenção Primária à Saúde (APS) sobre abordagem da sexualidade entre as pessoas idosas.

## MÉTODOS

### Aspectos éticos

Nesta pesquisa, foram considerados os aspectos éticos pertinentes às pesquisas envolvendo seres humanos, de acordo com a Resolução nº 466/12^(19)^, sendo aprovada pelo Comitê de Ética de Pesquisa da Universidade Federal da Paraíba. O Termo de Consentimento Livre e Esclarecido foi obtido de todos os indivíduos envolvidos no estudo por meio de documento escrito. Para manter o anonimato, as falas dos participantes foram identificadas ao longo do texto com a letra “P”, que representa os profissionais de saúde, seguida por um algarismo correspondente à ordem das falas (P01... P40).

### Tipo de estudo

Trata-se de pesquisa exploratória de abordagem qualitativa, que se concentra na compreensão da experiência humana como vivida, por meio da coleta e análise cuidadosa de materiais narrativos e subjetivos^(20)^. O estudo também foi fundamentado nos critérios do *COnsolidated criteria for REporting Qualitative research* (COREQ), recomendados para relatos de pesquisa realizados por meio de entrevistas ou grupos focais, com critérios bem definidos e estruturados em três domínios: caracterização e qualificação da equipe de pesquisa; desenho do estudo; e análise dos resultados^(21)^.

### Cenário do estudo

Esta pesquisa foi realizada nas Unidades Básicas de Saúde (UBS) do município de João Pessoa, Paraíba, entre outubro de 2018 e abril de 2019. O município conta com 93 UBS e 203 equipes de Saúde da Família (eSF) em seu território^(22)^.

### Fonte de dados

Foram entrevistados 20 médicos e 20 enfermeiros, com base nos seguintes critérios de inclusão: prestar assistência direta às pessoas idosas na APS no momento da coleta de dados e aceitar participar da pesquisa. A amostra foi, portanto, uma amostra por conveniência.

### Coleta e organização dos dados

A coleta de dados ocorreu por meio de entrevistas semiestruturadas gravadas, previamente agendadas com os profissionais de saúde. As entrevistas foram realizadas em uma sala privativa na própria UBS, fora do turno de trabalho dos participantes, para garantir a confidencialidade e minimizar interferências. Cada entrevista teve uma duração média de 30 minutos. O instrumento de coleta foi composto por duas partes: a primeira explorou dados sociodemográficos dos participantes, como idade, sexo, profissão e tempo de atuação; a segunda parte contemplou questões relacionadas à abordagem da sexualidade para pessoas idosas no atendimento cotidiano da eSF.

A pesquisa foi concluída considerando o intervalo de tempo proposto para a coleta de dados, a paridade numérica entre as duas classes profissionais, a qualidade do material, a ausência de informações novas e, consequentemente, a saturação dos dados.

### Análise dos dados

Os dados coletados pelas entrevistas foram analisados por meio da técnica de análise de conteúdo proposta por Bardin^(23)^, seguindo as seguintes etapas: constituição do *corpus* formado pelas 40 entrevistas, utilizando-se o critério de exaustividade; leitura flutuante e preparação do material (transcrição e leitura na íntegra das entrevistas gravadas); codificação (os recortes foram realizados por nível semântico, utilizando-se como unidade de registro a unidade temática, com a identificação dos(as) entrevistados(as) pela numeração P01-40); categorização, a qual seguiu a lógica semântica e de acordo com o objetivo do estudo.

Após a aplicação da técnica, os resultados revelaram cinco categorias temáticas, a saber: Descrições sobre sexualidade; Dificuldades para abordar a sexualidade; Importância de abordar a sexualidade entre os idosos; Profissional indicado para abordar a sexualidade; e Percepções dos idosos sobre a sexualidade segundo os profissionais de saúde.

O agrupamento e a categorização foram validados por três diferentes pesquisadores com *expertise* na técnica de análise de conteúdo e interpretação dos resultados.

## RESULTADOS

Participaram da pesquisa 40 profissionais de saúde, sendo 20 enfermeiros e 20 médicos, com a média de idade de 47 anos (mínimo 25 e máximo 70 anos) e tempo de profissão de 20 anos (mínimo 1 e máximo 46 anos).

Os resultados obtidos a partir da análise de conteúdo das entrevistas com médicos e enfermeiros que atuam na APS apontaram cinco categorias sobre como abordam a sexualidade com pessoas idosas.

### Categoria I - Descrições sobre sexualidade

Esta categoria foi formada pelas unidades de análise em que os profissionais de saúde (médicos e enfermeiros) falam sobre a sexualidade no atendimento às pessoas idosas. Verifica-se que a abordagem pode ser iniciada tanto pelo profissional de saúde quanto pela própria pessoa idosa.

Neste estudo, observou-se que a abordagem da sexualidade não ocorre de maneira sistemática, sendo mencionada especialmente durante a coleta do exame citopatológico, realizada pelos profissionais de enfermagem. Os médicos, por outro lado, não citaram essa prática em suas falas.

Outro momento em que a temática da sexualidade pode ser abordada é durante as reuniões grupais, conforme mencionado por alguns enfermeiros e médicos. Campanhas de conscientização global, como “Outubro Rosa” e “Novembro Azul”, que focam na prevenção do câncer de mama e de próstata, foram destacadas por esses profissionais como oportunidades para discutir assuntos relacionados à sexualidade entre pessoas idosas.

A abordagem (...) *nem frequente nem ocasional* (...) foi citada por poucos médicos e enfermeiros, como:

[...] *os pacientes chegam com essas queixas. Deveríamos questionar, mas, na prática, eles próprios tocam no assunto, então deveríamos perguntar, mas não ocorre isso* [...]. (P1)

[...] *no serviço, é feita a abordagem durante o citológico. As idosas procuram falar da dificuldade no ato sexual* [...]. (P4)

[...] *quase sempre a demanda vem dele* [...]. (P22)

[...] *esse tema a gente não fala, é muito trabalhoso e difícil, há muito preconceito, principalmente as idosas acham que não devem ter* [...]. (P24)

[...] *não abordo muito. Eles se queixam mais do que perguntamos* [...]. (P25)

Entretanto, nas consultas individuais, a maioria dos médicos e enfermeiros relatou que a sexualidade é abordada apenas mediante solicitação das próprias pessoas idosas. Destaca-se que alguns profissionais, tanto da enfermagem quanto da medicina, afirmaram que não discutem a sexualidade em nenhum momento. Do ponto de vista das pessoas idosas, é comum que falem sobre sexualidade entre si; no entanto, essa conversa ocorre de forma ocasional, segundo poucos enfermeiros e médicos. Outros mencionaram que essa abordagem costuma acontecer durante a coleta do exame citopatológico.

### Categoria II - Dificuldades para abordar a sexualidade

Esta categoria é formada por unidades de análise em que os profissionais de saúde descrevem desafios enfrentados para falarem sobre sexualidade com pessoas idosas. Nesse sentido, eles mencionam diversos obstáculos, como falta de treinamento e capacitação adequada, além da ausência de materiais apropriados para auxiliar na abordagem do tema.

Observam-se nas falas sentimentos de desconforto, preconceito e constrangimento, tanto por parte das pessoas idosas quanto por parte dos próprios profissionais de saúde, para lidar com o tema:

[...] *tenho dificuldade nesse assunto. Um treinamento seria bom para nos orientar, dar um direcionamento, se estamos certos, errados, como abordar da melhor maneira* [...]. (P7)

[...] *seria importante ter um material, um treinamento. São poucos que relatam, a maioria não fala mesmo, os homens procuram pouco* [...]. (P13)

[...] *tem tabu no assunto, mas tento perguntar naturalmente, acho que falta treinamento para ser mais natural, uma abordagem mais natural* [...]. (P27)

[...] *não teve na graduação treinamento para falar sobre esse assunto* [...]. (P40)

Observa-se que, ao falarem do preconceito social, das diferenças culturais e geracionais, os profissionais acrescentam a percepção de que o tema não é relevante em consultas de rotina, especialmente quando comparado às questões de saúde consideradas mais urgentes. Além disso, há a presença de crenças pessoais de alguns profissionais que influenciam a abordagem sobre o tema, dificultando a discussão da sexualidade no atendimento.

Nas falas dos profissionais, verifica-se a percepção de que as pessoas idosas sentem “desconforto, preconceito, tabu e constrangimento” ao abordar o assunto, conforme relatos tanto por médicos quanto por enfermeiros. Isso demonstra ser um dos principais obstáculos para a realização da abordagem sobre sexualidade durante o atendimento.

Os profissionais relataram que muitas pessoas idosas evitam falar sobre o tema por se sentirem envergonhadas ou por acreditarem que a sexualidade não deve ser discutida em sua faixa etária. Esse desconforto, segundo os profissionais, é agravado por preconceitos culturais que associam a sexualidade à juventude, fazendo com que as pessoas idosas se sintam inapropriadas ou julgadas ao falar sobre o assunto:

[...] *acho que há dificuldades, principalmente dos idosos, de eles se abrirem e falar* [...]. (P4)

[...] *há barreiras ainda, eles não falam tão naturalmente* [...]. (P18)

[...] *há muito preconceito principalmente por parte dos idosos. Acaba sendo difícil abordar* [...]. (P19)

[...] *como eles têm um tabu, ainda é um assunto fechado para eles. Acaba que eles não se protegem* [...]. (P21)

[...] *acho que ainda tem tabu, principalmente em relação aos idosos para os profissionais sobre o assunto* [...]. (P25)

[...]*percebo preconceito por parte dos mesmos. Não vejo dificuldades da minha parte* [...]. (P39)

Verifica-se que os profissionais destacam que o “constrangimento” pode gerar barreiras na comunicação, dificultando a criação de um ambiente seguro e acolhedor para que as pessoas idosas expressem suas preocupações ou necessidades sexuais. Alguns profissionais relataram que, mesmo quando tentam introduzir o tema, muitas vezes encontram resistência ou recusas para falar como uma forma de evitar causar mais desconforto à pessoa idosa.

Nas falas dos entrevistados, identificam-se unidades de análise em que ressaltam o “preconceito” por parte da sociedade em relação ao tema da sexualidade na velhice, considerado de difícil abordagem profissional e um estigma social. Esse aspecto se configura como uma das principais barreiras para essa abordagem, mesmo de forma natural e aberta, durante o atendimento à pessoa idosa.

Sabe-se que a sociedade ainda impõe restrições culturais ao associar a prática sexual às fases mais jovens da vida, reforçando a ideia de que a pessoa idosa não deve ou não pode ter uma vida sexual ativa:

[...]*há muito preconceito, também não sabemos lidar e abordar adequadamente sem ferir os princípios de alguns* [...]. (P4)

[...] *vivemos em um país de tabus, a abordagem é difícil, tem tabu no assunto* [...]. (P22)

[...] *acho que falta ser mais natural* [...]. (P27)

Observa-se, por parte dos profissionais, um desconforto maior tanto para eles quanto para as pessoas idosas ao falarem sobre sexualidade, especialmente quando a interação ocorre entre profissionais e pessoas idosas de sexos diferentes. Esse aspecto pode inibir a conversa, especialmente quando a pessoa idosa é mulher e o profissional de saúde é homem, ou vice-versa. Alguns entrevistados relataram que as pessoas idosas preferem discutir questões de sexualidade com profissionais de enfermagem do sexo feminino, pois se sentem mais à vontade. Por outro lado, os homens podem ter dificuldades em abordar a sexualidade com profissionais do sexo oposto, especialmente ao tratarem de questões relacionadas à impotência sexual:

[...]*não me sinto à vontade de falar sobre o assunto com os idosos homens* [...]. (P6)

[...] *as mulheres sentem mais dificuldades de falar com médicos homens* [...]. (P31)

[...]*eles são mais encabulados, porque eu acho que, como eu sou mulher, eles ficam com vergonha de falar sobre o assunto* [...]. (P38)

[...] *eu acho que, por eu ser mais nova, eu tive dificuldade tanto de ter naturalidade no assunto como percebi dificuldades da parte deles. Acaba que eles não se queixam para mim* [...]. (P15)

[...] *assunto é desconfortável. Sou de uma geração que é muito difícil falar, não só para os idosos* [...]. (P29)

[...] *não há nenhuma dificuldade, nenhuma, nem da parte dos profissionais, nem dos idosos. Os idosos participam, numa boa, das dinâmicas, aceitação total* [...]. (P5)

[...] *não tenho dificuldades de falar sobre essa abordagem pelo meu jeito de ser, sou muito brincalhona, a gente abre o assunto e eles dizem que não têm mais vontade ou que não conseguem fazer* [...]. (P37)

Neste sentido, alguns enfermeiros relataram não haver dificuldades em mencionar caso seja necessária a realização de uma atualização, em concordância com alguns médicos. Os enfermeiros ressaltaram uma preocupação com a diferença intergeracional, aspecto esse que não foi identificado nas falas dos médicos.

### Categoria III - Importância da abordar a sexualidade entre os idosos

Os profissionais destacaram a relevância do tema e a necessidade de realizar tal abordagem sobre a sexualidade entre as pessoas idosas de forma mais frequente. Neste sentido, todos os enfermeiros enfatizaram a importância de abordar a sexualidade para pessoas idosas, que deveria ser incluída na rotina. Outros profissionais expressaram que, apesar de acharem o assunto importante, a abordagem deveria ocorrer em grupos, palestras, campanhas ou quando solicitada pelas pessoas idosas. Os médicos consideraram relevante abordar o assunto, contudo, semelhante aos enfermeiros, a maioria mencionou que a abordagem deveria ser rotineira, enquanto alguns entendem que não seria importante. Observa-se que não houve consenso entre os profissionais:

[...] *sim, pois eles se entristecem mais ainda sem a vida sexual. É um somatório. Para a gente que tem entendimento, afeta, imagina para quem não tem* [...]. (P11)

[...] *acho importante demais. Hoje, o índice de IST tem aumentado muito. Eles ficam com vergonha de usar preservativo. Eles demoram para nos procurar quando têm sintomas, e temos idosos com doenças sexualmente transmissíveis, devendo ser avaliação rotineira* [...]. (P12)

[...] *acho interessante se abrir mais sobre esse assunto, pois pode ter vários problemas que estão sendo ocultados* [...]. (P13)

[...] *sim, importante demais, deveria ser rotina. Nós precisamos fazer esse questionamento* [...]. (P26)

[...] *muito importante, faz parte da vida, problemas de ereção, ansiedade, acaba virando uma bola de neve. Quando aborda, melhora a saúde mental* [...]. (P33)

A diferença entre os sexos também foi referida pelos entrevistados como uma barreira na abordagem da sexualidade entre as pessoas idosas. Médicos e enfermeiros relataram que, muitas vezes, homens e mulheres têm diferentes níveis de abertura e conforto ao falar sobre sua sexualidade, o que pode dificultar a abordagem do tema. Segundo os profissionais, os homens tendem a abordar mais diretamente questões relacionadas ao desempenho sexual, enquanto as mulheres, ao mencionarem o tema, costumam focar em aspectos emocionais ou na falta de desejo.

### Categoria IV - Profissionais indicados para abordar a sexualidade

Nesta categoria, os entrevistados apontaram os profissionais mais indicados para falar sobre sexualidade com as pessoas idosas durante o atendimento de saúde. Os enfermeiros, em sua maioria, destacaram que todos os profissionais de saúde devem proceder à referida abordagem. Para tanto, sugerem a necessidade de capacitar os profissionais para o atendimento. Apenas dois relataram que essa responsabilidade cabe aos médicos, enfermeiros e psicólogos. Quanto aos médicos, observou-se que 11 afirmaram que todos os profissionais de saúde devem falar sobre sexualidade; oito indicaram os médicos, enfermeiros e psicólogos; e apenas um entrevistado mencionou que somente os médicos devem falar sobre sexualidade com as pessoas idosas:

[...] *toda a equipe de profissionais, se tiver conhecimento e não tornar o assunto vulgar* [...]. (P3)

[...] *toda a equipe deveria ser treinada, os agentes de saúde também, pois tem muito idoso que possui um elo com os agentes, e eles trazem a queixa, os problemas* [...]. (P5)

[...] *a minha opinião é que todos sejam capacitados para tal, para não desfazer o que está sendo trabalhado: médicos, enfermeiros, dentistas, técnicos de enfermagem, agentes de saúde* [...]. (P23)

[...] *toda equipe deve abordar* [...]. (P25)

Verifica-se que os profissionais apontaram a falta de tempo nas consultas como um obstáculo relevante, já que muitos profissionais consideram que há pouco espaço para discussões sobre sexualidade em um atendimento voltado, majoritariamente, para questões físicas e crônicas. A maioria dos profissionais referiu enfrentar dificuldades em lidar com o tema, justificando a falta de treinamento, capacitação e recursos materiais como uma barreira significativa para abordar a sexualidade com as pessoas idosas.

### Categoria V - Percepções dos idosos sobre a sexualidade segundo os profissionais de saúde

Nesta categoria, observam-se unidades de análise dos profissionais abordando a importância de se falar sobre sexualidade com as pessoas idosas durante o atendimento de saúde. Os profissionais informam que as pessoas idosas demonstram preocupação com a “redução da libido”, conforme “falta de vontade” expressa nas consultas:

[...] *as idosas falam que os maridos querem e elas não querem mais* [...] *não sentem vontade, ou o contrário, elas querem e eles não querem.* (P2)

[...] *acham que as pessoas acham ridículo eles falarem sobre sexo.* (P12)

[...] *acham que não deveriam falar abertamente sobre o assunto* [...]. (P15)

[...] *eles reclamam muito do processo de envelhecimento, das dificuldades, do ressecamento* [...]. (P18)

[...] *eles pedem desculpas, muita vergonha, faz um arrodeio para falar. Eles acham que o desejo do sexo é errado, que ela tá safadinha, já falam que estão com vergonha* [...]. (P21)

*Eles acham que não têm mais idade* [...]. (P29)

*Muitos idosos já chegam falando da dispareunia, das dificuldades da menopausa que acabam atrapalhando a vida sexual* [...]. (P33)

Percebe-se uma preocupação dos entrevistados com o “preconceito” e as “dificuldades” associadas ao processo de envelhecimento no que diz respeito à sexualidade das pessoas idosas.

## DISCUSSÃO

Os resultados revelaram a proeminente dificuldade em abordar a temática da sexualidade entre as pessoas idosas no atendimento dos profissionais de saúde atuantes na APS, com destaque para as dificuldades relacionadas à formação profissional, valores pessoais, conflitos intergeracionais e construção social das pessoas idosas como assexuadas.

Tais dificuldades foram relatadas em outro estudo envolvendo profissionais da APS que apresentaram posicionamentos conservadores em relação à sexualidade entre pessoas idosas, mesmo demonstrando conhecimento sobre o assunto^(24)^. Destaca-se que as dificuldades encontradas neste estudo, como falta de conhecimento e treinamento sobre o tema, falta de tempo nas consultas e desconforto gerado pelo assunto, foram também identificados em outros estudos relacionados^(8,11,18,25)^.

Os profissionais de saúde entrevistados revelaram que abordar o tema da sexualidade entre pessoas idosas é difícil para a maioria deles: 80% dos médicos relataram essas dificuldades, assim como 70% dos enfermeiros. Ao analisar as principais dificuldades, destaca-se que 57,14% dos enfermeiros e 50% dos médicos mencionaram como dificuldades o desconforto, o preconceito e o constrangimento por parte das pessoas idosas. Esse achado foi corroborado por estudo realizado em Belém-PA, em 2017, sobre a percepção dos profissionais de saúde acerca da sexualidade na terceira idade, que indicou que as maiores dificuldades encontradas ao abordar o tema foram a “resistência dos idosos”, conforme relatado por 50% dos entrevistados^(26)^.

No que diz respeito às dificuldades relacionadas à falta de treinamento, preparo e capacitação no assunto, estas foram mencionadas por 57,14% dos enfermeiros e 37,5% dos médicos nesta pesquisa. Esses obstáculos também foram relatados em estudo com médicos residentes de saúde sexual e reprodutiva na comunidade, além de médicos residentes de obstetrícia e ginecologia na Inglaterra, que apontaram que as principais barreiras para abordar a saúde sexual na (peri)menopausa pelos médicos foram o desconforto percebido por parte do paciente e a falta de treinamento no currículo. Este estudo também mencionou a falta de tempo como uma barreira principal, que foi citada apenas por uma enfermeira entrevistada na nesta pesquisa^(14)^.

A falta de treinamento também foi mencionada em pesquisa realizada em São Paulo, que indicou que a maioria dos médicos não consegue abordar a questão da sexualidade de seus pacientes idosos com dor crônica devido a sentimentos de inaptidão técnica, além de falta de tempo e medo de constranger o paciente^(8)^. Estudo realizado com estudantes de enfermagem apontou que a sexualidade da pessoa idosa é vista como algo negativo e rotulado de estereótipos. As falas do estudo indicam uma maior dificuldade na abordagem do assunto com as pessoas idosas, fruto de uma cultura que os rotulam como assexuados e da formação deficitária dos profissionais de saúde^(26)^.

Outra dificuldade mencionada no presente estudo foi a diferença entre os sexos, relatada por 16,6% dos médicos e por 7,14% dos enfermeiros entrevistados. Essa percepção é corroborada por outra pesquisa realizada com os profissionais de saúde, na qual os entrevistados identificaram o sexo como um fator que interfere na discussão da sexualidade, com muitos homens se sentindo desconfortáveis ao falar sobre sexo com uma profissional da saúde do sexo feminino^(24)^. Ademais, em outro estudo, pessoas idosas do sexo feminino relataram sentir desconfortos ao realizar exame ginecológico com profissionais do sexo masculino, o que gera grande insegurança e desestímulo em procurar tais profissionais^(27)^.

No que diz respeito ao momento da abordagem da temática da sexualidade, médicos e enfermeiros destacam que a realizam durante o atendimento individual. No entanto, a maioria dos enfermeiros (65%) relatou que aborda o tema durante a coleta do exame citopatológico do colo do útero. Contudo, recente estudo brasileiro destacou que os profissionais de enfermagem falam pouco sobre o tema e precisam abordar a sexualidade sem julgamentos, segundo as pessoas idosas, visto que existem diferentes maneiras de encarar a sexualidade durante o processo de envelhecimento^(27)^.

Nesse sentido, é necessário enfatizar a importância de os profissionais estarem mais atentos e preparados para oferecer suporte à pessoa idosa sobre a temática da sexualidade, como demonstrado em outros estudos^(24,27,28)^. Eles devem se eximir de tabus, preconceitos e críticas, adotando posicionamentos mais adequadas diante dessa temática, visto que ainda se percebe um posicionamento conservador em relação à sexualidade e um despreparo dos profissionais de saúde^(17,27,28)^.

A entrevista com os médicos demonstrou que a abordagem sobre sexualidade é feita nas consultas de forma ocasional, priorizando quando é iniciada pelas pessoas idosas. Tal posicionamento, embora insuficiente, também é observado em outros estudos, como o de Oxford^(12)^, que identificou que 42,5% dos geriatras nunca perguntavam sobre a história sexual de seus pacientes e 57,5% o faziam apenas ocasionalmente. Em recente estudo realizado com 596 pessoas idosas nas cinco regiões brasileiras, evidenciou-se que 77,0% dos participantes nunca receberam orientações sobre sexualidade dos profissionais de saúde^(25)^. Outra pesquisa brasileira corrobora essa realidade, visto que apenas 17% a 32% dos homens e 19% a 29% das mulheres brasileiras receberam alguma orientação sobre sexualidade de profissionais médicos^(29)^.

Dessa forma, ao esperar que as pessoas idosas apresentem interesse pela abordagem de sua sexualidade, muitas oportunidades são perdidas. A literatura aponta que, mesmo na presença de disfunções que afetam gravemente a qualidade de vida, a maioria se sente desconfortável ou relutante em discutir a sexualidade com os profissionais de saúde, especialmente se tiver que iniciar as questões por conta própria^(18,24)^. Além disso, estudo revela que as pessoas idosas percebem que os profissionais de saúde não estão interessados ou não entendem a sexualidade dessa faixa etária e, por isso, evitam iniciar as discussões sobre o tema^(24)^. Em contrapartida, outra pesquisa demonstra que pessoas idosas que receberam alguma orientação sobre sexualidade dos profissionais de saúde vivenciam melhor a vida e o ato sexual^(30)^.

Apesar de, na prática clínica, a maioria da abordagem ser ocasional, todos os enfermeiros e 95% dos médicos relataram que é importante abordar o tema. Essa semelhança também observada em pesquisa na Inglaterra, em 2011, que evidenciou que 96,7% dos geriatras consideram importante gerenciar os problemas sexuais^(12)^.

As equipes de enfermagem e médica, na categoria profissional indicada para abordar a sexualidade, atribuíram a todos os profissionais de saúde a necessidade de proceder com a abordagem e de serem capacitados para o assunto. Estudo corroborou essa ideia ao demonstrar que todos os prestadores de cuidados de saúde, incluindo psicólogos, médicos (como os de cuidados primários, ginecologia, urologia, cuidados primários e oncologia), assistentes sociais, enfermeiros, farmacêuticos, terapeutas ocupacionais, educadores de saúde, diretores de lar de idosos e nutricionistas, podem potencialmente participar na modulação dos posicionamentos das pessoas idosas diante da sua própria vida sexual, tanto de forma direta quanto indireta^(17)^.

A falta de comunicação com os profissionais de saúde não apenas resulta em uma má gestão do tratamento, mas também favorece o risco de transmissão de infecções sexualmente transmissíveis (ISTs). Isso foi evidenciado em estudo transversal (n=101) com pessoas idosas, no qual 90% dos entrevistados relataram nunca ter recebido informações sobre o vírus da imunodeficiência humana (HIV) e 80% não receberam educação sobre ISTs. Como resultado, as pessoas idosas podem acreditar que não estão em risco de contrair uma doença sexualmente transmissível. Portanto, é evidente a necessidade de melhorar a comunicação entre profissionais e pessoas idosas, pois observa-se que os profissionais ainda mantêm posicionamentos conservadores em relação ao tema da sexualidade, além de demonstrarem posicionamentos negativos^(24)^.

Este estudo demonstra que uma área importante da atenção à saúde continua negligenciada, com a literatura indicando que a sexualidade é fundamental para o bem-estar de todos, incluindo as pessoas idosas^(25)^. É necessário que as pessoas sejam instruídas sobre as mudanças que ocorrem no funcionamento sexual à medida que envelhecem, bem como sobre as opções disponíveis para ajudá-las. Além disso, os profissionais de saúde precisam ser capacitados para aumentar a conscientização sobre a sexualidade no envelhecimento e aprimorar suas habilidades de comunicação^(18)^. O desafio consiste em desenvolver estratégias que permitam que a abordagem da sexualidade ocorra naturalmente como parte do cuidado à saúde das pessoas idosas.

### Limitações do estudo

As limitações desta pesquisa referem-se ao fato de que os resultados são baseados no conhecimento de apenas 40 profissionais de saúde que atuam em um único município do Nordeste brasileiro. A amostra é relativamente pequena e restrita a uma única localidade, o que limita a representatividade dos dados. Portanto, é importante realizar estudos com um maior número de profissionais de diferentes unidades de saúde para possibilitar generalizações mais robustas e precisas.

### Contribuições para as áreas da enfermagem e saúde

Explorar a sexualidade entre as pessoas idosas no cenário atual, caracterizado pelo crescente aumento da expectativa de vida da população brasileira e mundial, traz uma contribuição significativa para a desmistificação e ressignificação de conceitos arraigados na sociedade, como a ideia de que as pessoas idosas são assexuadas. Esses conceitos impactam diretamente a qualidade de vida dessas pessoas, influenciando sua saúde mental e física.

O estudo pode alertar gestores e profissionais de saúde, especialmente aqueles da APS - a porta de entrada do sistema de saúde brasileiro -, sobre a necessidade de incluir essa temática em suas capacitações e atendimento, tanto em grupos quanto de forma individual.

Os profissionais de saúde, incluindo enfermeiros, devem se preparar para a realidade do envelhecimento populacional e abordar a sexualidade no atendimento às pessoas idosas. Essa abordagem pode contribuir para a quebra de barreiras culturais e intergeracionais, além de promover uma assistência mais qualificada, ativa, integral e acolhedora. Este estudo busca trazer essas reflexões para a prática dos profissionais da APS e de outras áreas da rede de cuidado à saúde, visando estimular uma maior reflexão e divulgação sobre essa importante temática.

## CONSIDERAÇÕES FINAIS

O presente estudo procurou desvelar as vivências de profissionais na APS na abordagem da sexualidade com pessoas idosas, demonstrando que a abordagem é realizada de forma ocasional e sem proatividade. Os profissionais de saúde revelaram que, quando a abordagem acontece, percebe-se desconforto atrelado ao preconceito e falta de informação envolvendo a temática da sexualidade entre as pessoas idosas. Além disso, os profissionais evidenciam suas carências de treinamento para lidar com questões relacionadas à sexualidade.

A falta de comunicação entre os profissionais de saúde e os idosos negligencia as necessidades e favorece a aceitação dos estereótipos de assexuados e a naturalização da sexualidade como um assunto indesejável, fortalecendo as barreiras da assistência desta temática no processo de envelhecimento.

Nesse sentido, a abordagem da sexualidade na APS da pessoa idosa deve ser mais inclusiva e informada, uma vez que os profissionais de saúde desempenham um papel vital na desmistificação dos estereótipos associados à sexualidade das pessoas idosas e na promoção de uma vida sexual saudável e satisfatória. Essa mudança de paradigma não apenas beneficia as pessoas idosas, mas também enriquece o atendimento e fortalece a relação entre profissionais e pacientes.

Destacam-se como recomendações para minimizar tais concepções a necessidade de mais diálogos sobre sexualidade entre profissionais de saúde, visando uma melhor compreensão das necessidades das pessoas idosas, além de evitar a negligência em relação à saúde sexual e emocional, bem como a ausência de suporte na vivência da sexualidade. Portanto, é fundamental observar estratégias que minimizem estereótipos por meio da naturalização do tema entre pessoas idosas, buscando romper barreiras significativas na assistência à saúde. Superar esses desafios exige a implementação de ações voltadas à capacitação dos profissionais de saúde para abordar a sexualidade de forma mais aberta e respeitosa, reconhecendo a importância desse aspecto na vida das pessoas idosas.
